# [Retracted] Protective effect of *Xingnaojia* formulation on rats with brain and liver damage caused by chronic alcoholism 

**DOI:** 10.3892/etm.2025.12864

**Published:** 2025-04-07

**Authors:** Shuang Li, Su Wang, Zhi-Gang Guo, Ning Huang, Fan-Rong Zhao, Mo-Li Zhu, Li-Juan Ma, Jin-Ying Liang, Yu-Lin Zhang, Zhong-Lin Huang, Guang-Rui Wan

Exp Ther Med 10:1643–1652, 2015; DOI: 10.3892/etm.2015.2773

Following the publication of this article, a concerned reader drew to the Editor’s attention that, for the photomicrographs shown in Fig. 2 on p. 1647, panels F (the model group) and O (the high-dose XNJ group) appeared to be overlapping, albeit with changes in the blue/red ratio; in addition, panels F (model group) and L (low-dose XNJ group) also appeared to be overlapping, aganwith changes shown in the blue/red ratio. Furthermore, overlaps were also visible in the single fluorescence panels.

After having conducted an internal investigation, the Editor of *Experimental and Therapeutic Medicine* has reached the conclusion that this figure contained an unacceptable number of errors with respect to the compilation of the images/data. Therefore, on the grounds of a lack of confidence in the integrity of these data, the Editor has decided that the article should be retracted from the publication. The authors were asked for an explanation to account for these concerns, but the Editorial Office did not receive a reply. The Editor apologizes to the readership for any inconvenience caused, and thanks the interested reader for drawing this matter to our attention.

